# (2*E*)-1-[5-Methyl-1-(4-methyl­phen­yl)-1*H*-1,2,3-triazol-4-yl]-3-[4-(piperidin-1-yl)phen­yl]prop-2-en-1-one^1^

**DOI:** 10.1107/S1600536813008258

**Published:** 2013-04-05

**Authors:** Bakr F. Abdel-Wahab, Ehab Abdel-Latif, Seik Weng Ng, Edward R. T. Tiekink

**Affiliations:** a Applied Organic Chemistry Department, National Research Centre, Dokki, 12622 Giza, Egypt; b Department of Chemistry, University of Malaya, 50603 Kuala Lumpur, Malaysia; c Chemistry Department, Faculty of Science, King Abdulaziz University, PO Box 80203 Jeddah, Saudi Arabia

## Abstract

Two independent mol­ecules comprise the asymmetric unit of the title compound, C_24_H_26_N_4_O. The major difference between them is found in the relative orientation of the triazole-bound *p*-tolyl group which have the opposite sense of twist [N—N—C—C torsion angles = 55.8 (3) and −49.8 (3)°]. The chalcone residue is almost coplanar with the triazole ring [N—C—C—O and C—C—C—C torsion angles = −178.9 (2) and −178.5 (2)°, respectively; *cf*. 177.9 (3) and 168.5 (3)°, respectively, in the second mol­ecule]. The conformation about each C=C double bond is *E* and in each case the triazole methyl group is *syn* to the carbonyl O atom. In the crystal, mol­ecules aggregate into layers parallel to (-113). The first independent mol­ecule self-associates into a layer *via* C—H⋯O and C—H⋯π inter­actions. By contrast, layers comprising the second independent mol­ecule do not feature specific inter­actions between mol­ecules. The global crystal packing comprises alternating layers.

## Related literature

For the biological activities of triazole-based chalcone derivatives, see: Abdel-Wahab *et al.* (2012 ▶); Guantai *et al.* (2010 ▶). For a related structure, see: Abdel-Wahab *et al.* (2013 ▶).
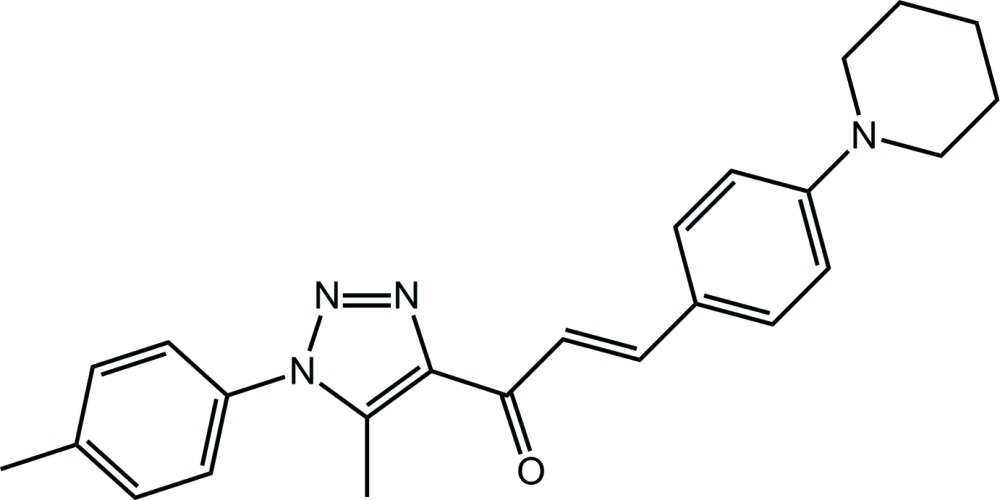


## Experimental

### 

#### Crystal data

C_24_H_26_N_4_O
*M* *_r_* = 386.49
Triclinic, 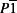

*a* = 12.9514 (16) Å
*b* = 13.1000 (13) Å
*c* = 13.3735 (14) Å
α = 77.666 (9)°
β = 74.123 (10)°
γ = 81.044 (9)°
*V* = 2120.6 (4) Å^3^
*Z* = 4
Mo *K*α radiation
μ = 0.08 mm^−1^
*T* = 295 K
0.50 × 0.40 × 0.30 mm

#### Data collection

Agilent SuperNova Dual diffractometer with an Atlas detector
Absorption correction: multi-scan (*CrysAlis PRO*; Agilent, 2011 ▶) *T*_min_ = 0.955, *T*_max_ = 1.000
17797 measured reflections
7458 independent reflections
4585 reflections with *I* > 2σ(*I*)
*R*_int_ = 0.034

#### Refinement

*R*[*F* ^2^ > 2σ(*F* ^2^)] = 0.058
*wR*(*F* ^2^) = 0.174
*S* = 1.03
7458 reflections
528 parameters
H-atom parameters constrained
Δρ_max_ = 0.19 e Å^−3^
Δρ_min_ = −0.19 e Å^−3^

### 

Data collection: *CrysAlis PRO* (Agilent, 2011 ▶); cell refinement: *CrysAlis PRO*; data reduction: *CrysAlis PRO*; program(s) used to solve structure: *SHELXS97* (Sheldrick, 2008 ▶); program(s) used to refine structure: *SHELXL97* (Sheldrick, 2008 ▶); molecular graphics: *ORTEP-3 for Windows* (Farrugia, 2012 ▶), *Qmol* (Gans & Shalloway, 2001 ▶) and *DIAMOND* (Brandenburg, 2006 ▶); software used to prepare material for publication: *publCIF* (Westrip, 2010 ▶).

## Supplementary Material

Crystal structure: contains datablock(s) global, I. DOI: 10.1107/S1600536813008258/hb7062sup1.cif

Structure factors: contains datablock(s) I. DOI: 10.1107/S1600536813008258/hb7062Isup2.hkl

Supplementary material file. DOI: 10.1107/S1600536813008258/hb7062Isup3.cml

Additional supplementary materials:  crystallographic information; 3D view; checkCIF report

## Figures and Tables

**Table 1:** Hydrogen-bond geometry (Å, °) *Cg*1 is the centroid of the C14–C19 benzene

*D*—H⋯*A*	*D*—H	H⋯*A*	*D*⋯*A*	*D*—H⋯*A*
C6—H6⋯O1^i^	0.93	2.51	3.415 (3)	165
C3—H3⋯*Cg*1^ii^	0.93	2.73	3.469 (3)	137

Symmetry codes: (i) 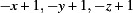; (ii) 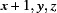.

**Fig. 1.**
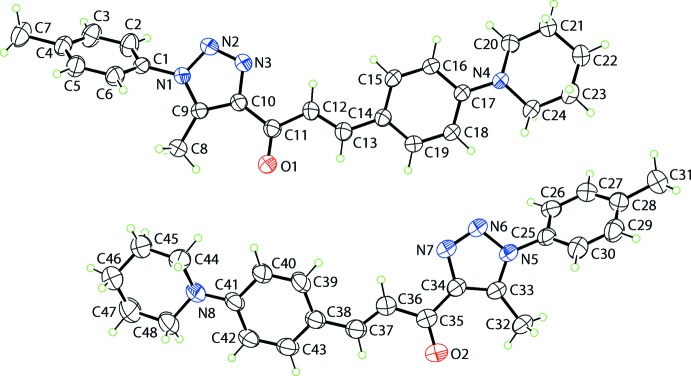
The molecular structure of (I) showing displacement ellipsoids at the 35% probability level.

**Fig. 2.**
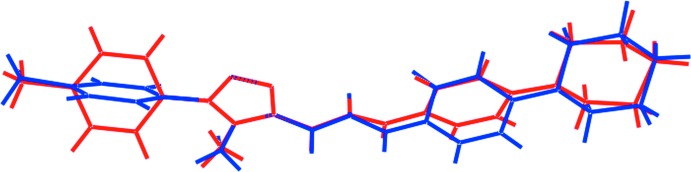
Overlay diagram of the two independent molecules comprising the asymmetric unit of (I). The O1- (red image) and O2-containing (blue) molecules have been superimposed so that the five-membered rings are coincident.

**Fig. 3.**
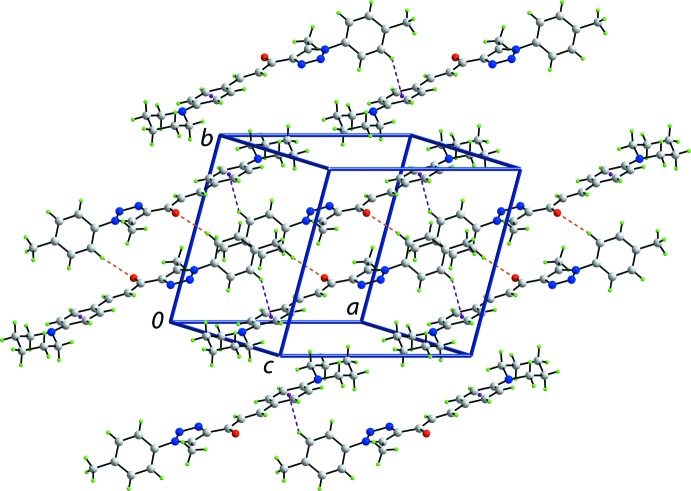
A view of the supramolecular layer in (I) comprising O1-containing molecules only, and sustained by C—H···O and C—H···π interactions, shown as orange and purple dashed lines, respectively.

**Fig. 4.**
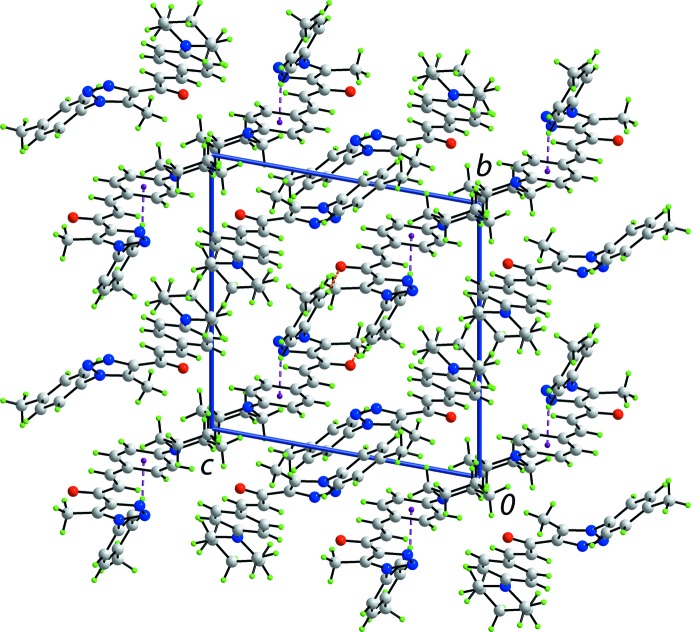
A view of the crystal packing in projection down the a axis, highlighting the alternating layers of O1- and O2-containing molecules. The C—H···O and C—H···π interactions are shown as orange and purple dashed lines, respectively.

## supplementary crystallographic information

### Comment

Triazole-based chalcone derivatives exhibit a range of biological activities (Abdel-Wahab *et al.*, 2012; Guantai *et al.*, 2010) and in this connection, the title compound was synthesized and characterized crystallographically.

In (I), Fig. 1, two independent molecules comprise the asymmetric unit. As illustrated in the overlay diagram, Fig. 2, variations exist in the relative orientations of the terminal substituents. In the central region of the molecule, the triazole-bound *p*-tolyl residue is twisted with respect to the five-membered ring, forming a dihedral angle of 56.88 (14)° [the comparable angle for the second independent molecule is 51.92 (16)°]. By contrast, the chalcone residue is co-planar as seen in the values of the N3—C11—C12—O1 and C11—C12—C13—C14 torsion angles of -178.9 (2) and -178.5 (2)°, respectively [*cf*. 177.9 (3) and 168.5 (3)°]. The conformation about each of C12═C13 and C36═C37 double bonds [1.329 (4) and 1.330 (4) Å] is *E*, and in each case the triazole-methyl is *syn* to the carbonyl-O atom. In these respects, the structure of (I) resembles closely that of a recently described derivative (Abdel-Wahab *et al.*, 2013). The major difference between the two molecules in (I) is found in the relative orientation of the triazole-bound *p*-tolyl groups. Although having similar dihedral angles, see above, the orientations of the rings have the opposite sense as seen in the value of the N2—N1—C1—C2 torsion angle of 55.8 (3)° *cf*. -49.8 (3)° for N6—N5—C25—C26. Each of the piperidinyl rings has a chair conformation.

In the crystal structure, centrosymmetrically related O1-containing molecules associate into dimers *via* C—H···O interactions and these are connected into a supramolecular layer, parallel to (-1 1 3), *via* C—H···π interactions, Fig. 3 and Table 1. The O2-containing molecules also assemble into a layer but without specific interactions between them. The global crystal packing comprises alternating layers of O1- and O2-containing layers with no specific interactions between them, Fig. 4.

### Experimental

The title compound was prepared following a reported method (Abdel-Wahab *et al.*, 2012). Yellow prisms were obtained from its DMF solution by slow evaporation at room temperature.

### Refinement

Carbon-bound H-atoms were placed in calculated positions (C—H = 0.93 to 0.97 Å) and were included in the refinement in the riding model approximation, with *U*_iso_(H) = 1.2–1.5*U*_equiv_(C).

### Crystal data

**Table:**

C_24_H_26_N_4_O	*Z* = 4
*M**_r_* = 386.49	*F*(000) = 824
Triclinic, *P*1	*D*_x_ = 1.211 Mg m^−^^3^
Hall symbol: -P 1	Mo *K*α radiation, λ = 0.71073 Å
*a* = 12.9514 (16) Å	Cell parameters from 3652 reflections
*b* = 13.1000 (13) Å	θ = 3.0–27.5°
*c* = 13.3735 (14) Å	µ = 0.08 mm^−^^1^
α = 77.666 (9)°	*T* = 295 K
β = 74.123 (10)°	Prism, yellow
γ = 81.044 (9)°	0.50 × 0.40 × 0.30 mm
*V* = 2120.6 (4) Å^3^	

### Data collection

**Table:**

Agilent SuperNova Dual diffractometer with an Atlas detector	7458 independent reflections
Radiation source: SuperNova (Mo) X-ray Source	4585 reflections with *I* > 2σ(*I*)
Mirror monochromator	*R*_int_ = 0.034
Detector resolution: 10.4041 pixels mm^-1^	θ_max_ = 25.0°, θ_min_ = 3.0°
ω scan	*h* = −14→15
Absorption correction: multi-scan (*CrysAlis PRO*; Agilent, 2011)	*k* = −15→15
*T*_min_ = 0.955, *T*_max_ = 1.000	*l* = −15→15
17797 measured reflections	

### Refinement

**Table:**

Refinement on *F*^2^	Secondary atom site location: difference Fourier map
Least-squares matrix: full	Hydrogen site location: inferred from neighbouring sites
*R*[*F*^2^ > 2σ(*F*^2^)] = 0.058	H-atom parameters constrained
*wR*(*F*^2^) = 0.174	*w* = 1/[σ^2^(*F*_o_^2^) + (0.0637*P*)^2^ + 0.5896*P*] where *P* = (*F*_o_^2^ + 2*F*_c_^2^)/3
*S* = 1.03	(Δ/σ)_max_ = 0.001
7458 reflections	Δρ_max_ = 0.19 e Å^−^^3^
528 parameters	Δρ_min_ = −0.19 e Å^−^^3^
0 restraints	Extinction correction: *SHELXL97* (Sheldrick, 2008), Fc^*^=kFc[1+0.001xFc^2^λ^3^/sin(2θ)]^-1/4^
Primary atom site location: structure-invariant direct methods	Extinction coefficient: 0.0048 (8)

### Special details

**Table:**

Geometry. All e.s.d.'s (except the e.s.d. in the dihedral angle between two l.s. planes) are estimated using the full covariance matrix. The cell e.s.d.'s are taken into account individually in the estimation of e.s.d.'s in distances, angles and torsion angles; correlations between e.s.d.'s in cell parameters are only used when they are defined by crystal symmetry. An approximate (isotropic) treatment of cell e.s.d.'s is used for estimating e.s.d.'s involving l.s. planes.
Refinement. Refinement of *F*^2^ against ALL reflections. The weighted *R*-factor *wR* and goodness of fit *S* are based on *F*^2^, conventional *R*-factors *R* are based on *F*, with *F* set to zero for negative *F*^2^. The threshold expression of *F*^2^ > σ(*F*^2^) is used only for calculating *R*-factors(gt) *etc*. and is not relevant to the choice of reflections for refinement. *R*-factors based on *F*^2^ are statistically about twice as large as those based on *F*, and *R*- factors based on ALL data will be even larger.

### Fractional atomic coordinates and isotropic or equivalent isotropic displacement parameters (Å^2^)

**Table:**

	*x*	*y*	*z*	*U*_iso_*/*U*_eq_	
O1	0.44643 (15)	0.32264 (15)	0.48227 (14)	0.0724 (5)	
O2	0.04267 (17)	0.20058 (17)	0.10647 (15)	0.0886 (6)	
N1	0.64525 (16)	0.39564 (16)	0.65410 (15)	0.0563 (5)	
N2	0.5765 (2)	0.3624 (2)	0.74989 (16)	0.0758 (7)	
N3	0.49501 (19)	0.33089 (19)	0.73114 (16)	0.0716 (6)	
N4	−0.14649 (16)	0.10222 (17)	0.86378 (15)	0.0592 (6)	
N5	−0.14790 (18)	0.11974 (17)	0.41775 (16)	0.0632 (6)	
N6	−0.0748 (2)	0.1474 (2)	0.46206 (18)	0.0782 (7)	
N7	0.0042 (2)	0.1822 (2)	0.38478 (18)	0.0754 (7)	
N8	0.6530 (2)	0.3801 (2)	0.09660 (18)	0.0793 (7)	
C1	0.74396 (19)	0.43353 (19)	0.65210 (18)	0.0533 (6)	
C2	0.8143 (2)	0.3704 (2)	0.7049 (2)	0.0764 (8)	
H2	0.7979	0.3038	0.7416	0.092*	
C3	0.9086 (2)	0.4059 (2)	0.7033 (3)	0.0794 (9)	
H3	0.9556	0.3628	0.7397	0.095*	
C4	0.9361 (2)	0.5039 (2)	0.6493 (2)	0.0625 (7)	
C5	0.8635 (2)	0.5658 (2)	0.5981 (2)	0.0708 (8)	
H5	0.8796	0.6324	0.5613	0.085*	
C6	0.7680 (2)	0.5322 (2)	0.5997 (2)	0.0651 (7)	
H6	0.7198	0.5761	0.5653	0.078*	
C7	1.0401 (2)	0.5407 (3)	0.6491 (3)	0.0925 (10)	
H7A	1.0950	0.4823	0.6486	0.139*	
H7B	1.0304	0.5702	0.7113	0.139*	
H7C	1.0614	0.5932	0.5874	0.139*	
C8	0.6648 (2)	0.4071 (2)	0.46175 (19)	0.0723 (8)	
H8A	0.6801	0.4791	0.4437	0.108*	
H8B	0.6211	0.3954	0.4185	0.108*	
H8C	0.7312	0.3620	0.4500	0.108*	
C9	0.60606 (19)	0.38366 (19)	0.57457 (17)	0.0530 (6)	
C10	0.5096 (2)	0.34268 (19)	0.62391 (18)	0.0561 (6)	
C11	0.4303 (2)	0.3127 (2)	0.5790 (2)	0.0582 (7)	
C12	0.3326 (2)	0.2737 (2)	0.6525 (2)	0.0613 (7)	
H12	0.3218	0.2745	0.7240	0.074*	
C13	0.2582 (2)	0.23702 (19)	0.6219 (2)	0.0585 (7)	
H13	0.2728	0.2359	0.5501	0.070*	
C14	0.15793 (19)	0.19881 (18)	0.68588 (18)	0.0531 (6)	
C15	0.1231 (2)	0.19481 (19)	0.79543 (19)	0.0576 (6)	
H15	0.1676	0.2154	0.8299	0.069*	
C16	0.0259 (2)	0.16163 (19)	0.85315 (19)	0.0576 (6)	
H16	0.0059	0.1609	0.9256	0.069*	
C17	−0.04496 (19)	0.12836 (18)	0.80569 (17)	0.0513 (6)	
C18	−0.0095 (2)	0.13069 (19)	0.69667 (18)	0.0571 (6)	
H18	−0.0528	0.1082	0.6622	0.069*	
C19	0.0881 (2)	0.16548 (19)	0.63936 (19)	0.0586 (7)	
H19	0.1082	0.1668	0.5668	0.070*	
C20	−0.1592 (2)	0.0469 (3)	0.97295 (19)	0.0757 (8)	
H20A	−0.1140	0.0745	1.0057	0.091*	
H20B	−0.1349	−0.0270	0.9730	0.091*	
C21	−0.2732 (2)	0.0575 (3)	1.0365 (2)	0.0845 (9)	
H21A	−0.2943	0.1302	1.0449	0.101*	
H21B	−0.2782	0.0151	1.1063	0.101*	
C22	−0.3501 (3)	0.0239 (3)	0.9860 (2)	0.0910 (10)	
H22A	−0.4239	0.0395	1.0252	0.109*	
H22B	−0.3367	−0.0513	0.9872	0.109*	
C23	−0.3339 (2)	0.0817 (3)	0.8733 (2)	0.0828 (9)	
H23A	−0.3780	0.0547	0.8388	0.099*	
H23B	−0.3583	0.1556	0.8734	0.099*	
C24	−0.2181 (2)	0.0711 (2)	0.8111 (2)	0.0673 (7)	
H24A	−0.1966	−0.0014	0.8020	0.081*	
H24B	−0.2112	0.1145	0.7416	0.081*	
C25	−0.2421 (2)	0.0763 (2)	0.4871 (2)	0.0623 (7)	
C26	−0.2301 (2)	−0.0049 (2)	0.5683 (2)	0.0728 (8)	
H26	−0.1617	−0.0335	0.5759	0.087*	
C27	−0.3209 (3)	−0.0434 (3)	0.6385 (2)	0.0871 (9)	
H27	−0.3128	−0.0983	0.6937	0.105*	
C28	−0.4233 (3)	−0.0031 (3)	0.6294 (2)	0.0863 (9)	
C29	−0.4326 (3)	0.0774 (3)	0.5458 (3)	0.0850 (9)	
H29	−0.5009	0.1046	0.5369	0.102*	
C30	−0.3432 (2)	0.1182 (2)	0.4754 (2)	0.0743 (8)	
H30	−0.3510	0.1735	0.4205	0.089*	
C31	−0.5221 (3)	−0.0425 (4)	0.7095 (3)	0.1369 (17)	
H31A	−0.5179	−0.0379	0.7790	0.205*	
H31B	−0.5258	−0.1143	0.7063	0.205*	
H31C	−0.5854	−0.0003	0.6943	0.205*	
C32	−0.1717 (2)	0.1099 (2)	0.2409 (2)	0.0773 (8)	
H32A	−0.2068	0.0477	0.2749	0.116*	
H32B	−0.1215	0.0977	0.1759	0.116*	
H32C	−0.2248	0.1670	0.2261	0.116*	
C33	−0.1133 (2)	0.1368 (2)	0.3113 (2)	0.0603 (7)	
C34	−0.0161 (2)	0.1772 (2)	0.2913 (2)	0.0625 (7)	
C35	0.0615 (2)	0.2088 (2)	0.1895 (2)	0.0678 (7)	
C36	0.1605 (2)	0.2454 (2)	0.1946 (2)	0.0746 (8)	
H36	0.1645	0.2592	0.2588	0.089*	
C37	0.2453 (3)	0.2599 (2)	0.1124 (2)	0.0728 (8)	
H37	0.2372	0.2484	0.0488	0.087*	
C38	0.3481 (2)	0.2913 (2)	0.1093 (2)	0.0696 (8)	
C39	0.3716 (3)	0.3167 (3)	0.1962 (2)	0.0944 (11)	
H39	0.3183	0.3134	0.2592	0.113*	
C40	0.4691 (3)	0.3462 (3)	0.1931 (2)	0.0935 (10)	
H40	0.4808	0.3613	0.2537	0.112*	
C41	0.5526 (2)	0.3539 (2)	0.0993 (2)	0.0685 (8)	
C42	0.5291 (3)	0.3297 (2)	0.0129 (2)	0.0785 (9)	
H42	0.5815	0.3347	−0.0508	0.094*	
C43	0.4311 (3)	0.2986 (2)	0.0178 (2)	0.0778 (9)	
H43	0.4200	0.2819	−0.0424	0.093*	
C44	0.6601 (3)	0.4424 (3)	0.1705 (3)	0.0968 (10)	
H44A	0.6182	0.4131	0.2399	0.116*	
H44B	0.6268	0.5128	0.1509	0.116*	
C45	0.7688 (3)	0.4503 (4)	0.1784 (3)	0.1224 (14)	
H45A	0.7938	0.3847	0.2185	0.147*	
H45B	0.7651	0.5054	0.2179	0.147*	
C46	0.8496 (3)	0.4727 (3)	0.0757 (3)	0.1112 (12)	
H46A	0.8376	0.5460	0.0440	0.133*	
H46B	0.9215	0.4602	0.0874	0.133*	
C47	0.8415 (3)	0.4057 (4)	0.0032 (3)	0.1300 (15)	
H47A	0.8833	0.4328	−0.0671	0.156*	
H47B	0.8746	0.3357	0.0249	0.156*	
C48	0.7316 (3)	0.3969 (3)	−0.0033 (3)	0.1035 (12)	
H48A	0.7073	0.4607	−0.0463	0.124*	
H48B	0.7346	0.3392	−0.0393	0.124*	

### Atomic displacement parameters (Å^2^)

**Table:**

	*U*^11^	*U*^22^	*U*^33^	*U*^12^	*U*^13^	*U*^23^
O1	0.0622 (12)	0.0989 (14)	0.0584 (11)	−0.0187 (10)	−0.0194 (9)	−0.0062 (10)
O2	0.0927 (16)	0.1176 (17)	0.0638 (12)	−0.0174 (12)	−0.0295 (11)	−0.0160 (11)
N1	0.0479 (13)	0.0748 (14)	0.0471 (11)	−0.0125 (10)	−0.0108 (10)	−0.0096 (10)
N2	0.0639 (16)	0.1163 (19)	0.0481 (12)	−0.0275 (14)	−0.0069 (11)	−0.0129 (12)
N3	0.0569 (15)	0.1061 (18)	0.0527 (13)	−0.0247 (13)	−0.0074 (11)	−0.0127 (12)
N4	0.0518 (13)	0.0839 (14)	0.0482 (11)	−0.0194 (11)	−0.0175 (10)	−0.0100 (10)
N5	0.0628 (15)	0.0776 (14)	0.0587 (13)	−0.0101 (11)	−0.0239 (11)	−0.0189 (11)
N6	0.0723 (17)	0.1110 (19)	0.0635 (14)	−0.0238 (14)	−0.0235 (13)	−0.0232 (13)
N7	0.0757 (17)	0.0997 (18)	0.0625 (14)	−0.0223 (13)	−0.0232 (13)	−0.0221 (13)
N8	0.0688 (18)	0.1040 (19)	0.0600 (14)	−0.0185 (14)	0.0037 (12)	−0.0223 (13)
C1	0.0469 (15)	0.0683 (16)	0.0485 (13)	−0.0071 (12)	−0.0140 (11)	−0.0153 (12)
C2	0.067 (2)	0.0680 (17)	0.098 (2)	−0.0094 (14)	−0.0361 (17)	−0.0012 (15)
C3	0.064 (2)	0.079 (2)	0.106 (2)	0.0007 (15)	−0.0438 (17)	−0.0145 (17)
C4	0.0486 (16)	0.0721 (17)	0.0746 (17)	−0.0040 (13)	−0.0175 (13)	−0.0288 (14)
C5	0.0681 (19)	0.0701 (17)	0.0785 (18)	−0.0174 (14)	−0.0256 (15)	−0.0055 (14)
C6	0.0594 (18)	0.0719 (17)	0.0664 (16)	−0.0079 (13)	−0.0261 (14)	−0.0033 (14)
C7	0.065 (2)	0.106 (2)	0.123 (3)	−0.0109 (17)	−0.0322 (19)	−0.045 (2)
C8	0.0708 (19)	0.098 (2)	0.0507 (15)	−0.0301 (16)	−0.0099 (13)	−0.0102 (14)
C9	0.0489 (15)	0.0654 (15)	0.0455 (13)	−0.0068 (12)	−0.0126 (11)	−0.0099 (11)
C10	0.0470 (15)	0.0700 (16)	0.0492 (14)	−0.0074 (12)	−0.0093 (12)	−0.0087 (11)
C11	0.0496 (16)	0.0676 (16)	0.0551 (15)	−0.0038 (12)	−0.0129 (12)	−0.0078 (12)
C12	0.0469 (16)	0.0779 (17)	0.0584 (15)	−0.0111 (13)	−0.0115 (13)	−0.0098 (13)
C13	0.0519 (16)	0.0645 (15)	0.0586 (14)	−0.0072 (12)	−0.0136 (12)	−0.0092 (12)
C14	0.0478 (15)	0.0574 (14)	0.0547 (14)	−0.0057 (11)	−0.0149 (12)	−0.0088 (11)
C15	0.0520 (16)	0.0717 (16)	0.0551 (14)	−0.0122 (12)	−0.0230 (12)	−0.0077 (12)
C16	0.0545 (16)	0.0766 (17)	0.0461 (13)	−0.0120 (13)	−0.0179 (12)	−0.0098 (12)
C17	0.0494 (15)	0.0590 (14)	0.0490 (13)	−0.0096 (11)	−0.0172 (12)	−0.0078 (11)
C18	0.0572 (17)	0.0695 (16)	0.0523 (14)	−0.0158 (13)	−0.0197 (13)	−0.0127 (12)
C19	0.0604 (17)	0.0702 (16)	0.0479 (13)	−0.0135 (13)	−0.0139 (13)	−0.0111 (12)
C20	0.0629 (19)	0.110 (2)	0.0531 (15)	−0.0232 (16)	−0.0166 (14)	−0.0003 (15)
C21	0.062 (2)	0.132 (3)	0.0629 (17)	−0.0335 (18)	−0.0085 (15)	−0.0159 (17)
C22	0.066 (2)	0.133 (3)	0.078 (2)	−0.0405 (19)	−0.0055 (16)	−0.0238 (19)
C23	0.0576 (19)	0.121 (3)	0.083 (2)	−0.0231 (17)	−0.0222 (16)	−0.0300 (18)
C24	0.0605 (18)	0.0876 (19)	0.0635 (16)	−0.0224 (14)	−0.0226 (14)	−0.0143 (14)
C25	0.0587 (18)	0.0760 (17)	0.0607 (15)	−0.0068 (14)	−0.0169 (14)	−0.0279 (14)
C26	0.071 (2)	0.095 (2)	0.0564 (16)	−0.0062 (16)	−0.0221 (15)	−0.0162 (15)
C27	0.085 (2)	0.116 (3)	0.0623 (18)	−0.025 (2)	−0.0186 (17)	−0.0103 (17)
C28	0.069 (2)	0.128 (3)	0.0697 (19)	−0.0224 (19)	−0.0068 (17)	−0.0401 (19)
C29	0.061 (2)	0.104 (2)	0.101 (2)	0.0009 (17)	−0.0214 (19)	−0.047 (2)
C30	0.067 (2)	0.0739 (18)	0.089 (2)	−0.0024 (15)	−0.0246 (17)	−0.0250 (15)
C31	0.090 (3)	0.230 (5)	0.090 (3)	−0.059 (3)	0.001 (2)	−0.029 (3)
C32	0.082 (2)	0.092 (2)	0.0730 (18)	−0.0178 (16)	−0.0350 (16)	−0.0202 (15)
C33	0.0640 (18)	0.0640 (16)	0.0604 (16)	−0.0001 (13)	−0.0261 (13)	−0.0185 (12)
C34	0.0636 (18)	0.0677 (16)	0.0620 (16)	−0.0058 (13)	−0.0230 (14)	−0.0154 (13)
C35	0.074 (2)	0.0694 (17)	0.0645 (17)	−0.0038 (14)	−0.0247 (15)	−0.0140 (14)
C36	0.076 (2)	0.086 (2)	0.0659 (17)	−0.0157 (16)	−0.0156 (16)	−0.0179 (15)
C37	0.083 (2)	0.0752 (18)	0.0606 (17)	−0.0128 (16)	−0.0164 (16)	−0.0121 (14)
C38	0.079 (2)	0.0726 (18)	0.0556 (16)	−0.0137 (15)	−0.0100 (15)	−0.0130 (13)
C39	0.084 (3)	0.133 (3)	0.0667 (19)	−0.045 (2)	0.0155 (17)	−0.0390 (18)
C40	0.090 (3)	0.134 (3)	0.0633 (18)	−0.045 (2)	0.0076 (17)	−0.0426 (18)
C41	0.071 (2)	0.0749 (18)	0.0531 (15)	−0.0131 (15)	0.0015 (14)	−0.0151 (13)
C42	0.072 (2)	0.106 (2)	0.0505 (16)	−0.0104 (17)	−0.0015 (15)	−0.0159 (15)
C43	0.084 (2)	0.095 (2)	0.0521 (16)	−0.0065 (17)	−0.0126 (15)	−0.0154 (14)
C44	0.083 (3)	0.134 (3)	0.080 (2)	−0.023 (2)	−0.0102 (18)	−0.037 (2)
C45	0.078 (3)	0.207 (5)	0.087 (2)	−0.044 (3)	−0.007 (2)	−0.033 (3)
C46	0.088 (3)	0.153 (3)	0.090 (2)	−0.039 (2)	−0.008 (2)	−0.016 (2)
C47	0.086 (3)	0.201 (5)	0.099 (3)	−0.044 (3)	0.019 (2)	−0.053 (3)
C48	0.091 (3)	0.139 (3)	0.077 (2)	−0.042 (2)	0.0142 (19)	−0.034 (2)

### Geometric parameters (Å, º)

**Table:**

O1—C11	1.234 (3)	C21—H21B	0.9700
O2—C35	1.230 (3)	C22—C23	1.510 (4)
N1—C9	1.343 (3)	C22—H22A	0.9700
N1—N2	1.372 (3)	C22—H22B	0.9700
N1—C1	1.433 (3)	C23—C24	1.505 (4)
N2—N3	1.293 (3)	C23—H23A	0.9700
N3—C10	1.372 (3)	C23—H23B	0.9700
N4—C17	1.385 (3)	C24—H24A	0.9700
N4—C24	1.456 (3)	C24—H24B	0.9700
N4—C20	1.462 (3)	C25—C26	1.373 (4)
N5—C33	1.350 (3)	C25—C30	1.375 (4)
N5—N6	1.372 (3)	C26—C27	1.377 (4)
N5—C25	1.429 (3)	C26—H26	0.9300
N6—N7	1.300 (3)	C27—C28	1.377 (4)
N7—C34	1.363 (3)	C27—H27	0.9300
N8—C41	1.386 (4)	C28—C29	1.380 (4)
N8—C48	1.439 (4)	C28—C31	1.507 (4)
N8—C44	1.438 (4)	C29—C30	1.377 (4)
C1—C6	1.371 (3)	C29—H29	0.9300
C1—C2	1.371 (3)	C30—H30	0.9300
C2—C3	1.366 (4)	C31—H31A	0.9600
C2—H2	0.9300	C31—H31B	0.9600
C3—C4	1.379 (4)	C31—H31C	0.9600
C3—H3	0.9300	C32—C33	1.484 (4)
C4—C5	1.374 (4)	C32—H32A	0.9600
C4—C7	1.499 (4)	C32—H32B	0.9600
C5—C6	1.368 (4)	C32—H32C	0.9600
C5—H5	0.9300	C33—C34	1.377 (4)
C6—H6	0.9300	C34—C35	1.474 (4)
C7—H7A	0.9600	C35—C36	1.460 (4)
C7—H7B	0.9600	C36—C37	1.329 (4)
C7—H7C	0.9600	C36—H36	0.9300
C8—C9	1.482 (3)	C37—C38	1.441 (4)
C8—H8A	0.9600	C37—H37	0.9300
C8—H8B	0.9600	C38—C43	1.387 (4)
C8—H8C	0.9600	C38—C39	1.395 (4)
C9—C10	1.375 (3)	C39—C40	1.365 (4)
C10—C11	1.461 (4)	C39—H39	0.9300
C11—C12	1.462 (3)	C40—C41	1.411 (4)
C12—C13	1.330 (4)	C40—H40	0.9300
C12—H12	0.9300	C41—C42	1.380 (4)
C13—C14	1.443 (3)	C42—C43	1.374 (4)
C13—H13	0.9300	C42—H42	0.9300
C14—C19	1.394 (3)	C43—H43	0.9300
C14—C15	1.402 (3)	C44—C45	1.460 (4)
C15—C16	1.365 (3)	C44—H44A	0.9700
C15—H15	0.9300	C44—H44B	0.9700
C16—C17	1.413 (3)	C45—C46	1.485 (4)
C16—H16	0.9300	C45—H45A	0.9700
C17—C18	1.399 (3)	C45—H45B	0.9700
C18—C19	1.376 (3)	C46—C47	1.474 (5)
C18—H18	0.9300	C46—H46A	0.9700
C19—H19	0.9300	C46—H46B	0.9700
C20—C21	1.490 (4)	C47—C48	1.472 (5)
C20—H20A	0.9700	C47—H47A	0.9700
C20—H20B	0.9700	C47—H47B	0.9700
C21—C22	1.508 (4)	C48—H48A	0.9700
C21—H21A	0.9700	C48—H48B	0.9700
			
C9—N1—N2	110.6 (2)	C22—C23—H23B	109.1
C9—N1—C1	130.4 (2)	H23A—C23—H23B	107.8
N2—N1—C1	118.9 (2)	N4—C24—C23	111.7 (2)
N3—N2—N1	107.3 (2)	N4—C24—H24A	109.3
N2—N3—C10	109.0 (2)	C23—C24—H24A	109.3
C17—N4—C24	119.2 (2)	N4—C24—H24B	109.3
C17—N4—C20	118.6 (2)	C23—C24—H24B	109.3
C24—N4—C20	112.7 (2)	H24A—C24—H24B	107.9
C33—N5—N6	110.8 (2)	C26—C25—C30	120.5 (3)
C33—N5—C25	131.2 (2)	C26—C25—N5	118.8 (2)
N6—N5—C25	118.0 (2)	C30—C25—N5	120.6 (3)
N7—N6—N5	107.0 (2)	C25—C26—C27	119.0 (3)
N6—N7—C34	109.1 (2)	C25—C26—H26	120.5
C41—N8—C48	118.8 (3)	C27—C26—H26	120.5
C41—N8—C44	118.5 (2)	C28—C27—C26	122.0 (3)
C48—N8—C44	114.6 (3)	C28—C27—H27	119.0
C6—C1—C2	119.7 (2)	C26—C27—H27	119.0
C6—C1—N1	121.0 (2)	C27—C28—C29	117.6 (3)
C2—C1—N1	119.3 (2)	C27—C28—C31	121.5 (3)
C3—C2—C1	119.6 (3)	C29—C28—C31	120.9 (3)
C3—C2—H2	120.2	C30—C29—C28	121.5 (3)
C1—C2—H2	120.2	C30—C29—H29	119.2
C2—C3—C4	121.9 (3)	C28—C29—H29	119.2
C2—C3—H3	119.0	C25—C30—C29	119.3 (3)
C4—C3—H3	119.0	C25—C30—H30	120.3
C5—C4—C3	117.1 (3)	C29—C30—H30	120.3
C5—C4—C7	122.2 (3)	C28—C31—H31A	109.5
C3—C4—C7	120.7 (3)	C28—C31—H31B	109.5
C6—C5—C4	121.9 (3)	H31A—C31—H31B	109.5
C6—C5—H5	119.0	C28—C31—H31C	109.5
C4—C5—H5	119.0	H31A—C31—H31C	109.5
C5—C6—C1	119.6 (2)	H31B—C31—H31C	109.5
C5—C6—H6	120.2	C33—C32—H32A	109.5
C1—C6—H6	120.2	C33—C32—H32B	109.5
C4—C7—H7A	109.5	H32A—C32—H32B	109.5
C4—C7—H7B	109.5	C33—C32—H32C	109.5
H7A—C7—H7B	109.5	H32A—C32—H32C	109.5
C4—C7—H7C	109.5	H32B—C32—H32C	109.5
H7A—C7—H7C	109.5	N5—C33—C34	104.0 (2)
H7B—C7—H7C	109.5	N5—C33—C32	123.7 (3)
C9—C8—H8A	109.5	C34—C33—C32	132.2 (3)
C9—C8—H8B	109.5	N7—C34—C33	109.1 (2)
H8A—C8—H8B	109.5	N7—C34—C35	121.3 (3)
C9—C8—H8C	109.5	C33—C34—C35	129.6 (3)
H8A—C8—H8C	109.5	O2—C35—C36	123.5 (3)
H8B—C8—H8C	109.5	O2—C35—C34	120.0 (3)
N1—C9—C10	104.5 (2)	C36—C35—C34	116.5 (3)
N1—C9—C8	123.4 (2)	C37—C36—C35	123.4 (3)
C10—C9—C8	132.0 (2)	C37—C36—H36	118.3
N3—C10—C9	108.6 (2)	C35—C36—H36	118.3
N3—C10—C11	121.3 (2)	C36—C37—C38	128.2 (3)
C9—C10—C11	130.1 (2)	C36—C37—H37	115.9
O1—C11—C10	119.9 (2)	C38—C37—H37	115.9
O1—C11—C12	122.6 (2)	C43—C38—C39	115.2 (3)
C10—C11—C12	117.5 (2)	C43—C38—C37	121.5 (3)
C13—C12—C11	123.0 (2)	C39—C38—C37	123.3 (3)
C13—C12—H12	118.5	C40—C39—C38	123.1 (3)
C11—C12—H12	118.5	C40—C39—H39	118.4
C12—C13—C14	128.3 (2)	C38—C39—H39	118.4
C12—C13—H13	115.9	C39—C40—C41	121.0 (3)
C14—C13—H13	115.9	C39—C40—H40	119.5
C19—C14—C15	116.0 (2)	C41—C40—H40	119.5
C19—C14—C13	120.4 (2)	C42—C41—N8	122.7 (3)
C15—C14—C13	123.6 (2)	C42—C41—C40	116.0 (3)
C16—C15—C14	122.1 (2)	N8—C41—C40	121.3 (3)
C16—C15—H15	118.9	C43—C42—C41	122.3 (3)
C14—C15—H15	118.9	C43—C42—H42	118.8
C15—C16—C17	121.7 (2)	C41—C42—H42	118.8
C15—C16—H16	119.1	C42—C43—C38	122.4 (3)
C17—C16—H16	119.1	C42—C43—H43	118.8
N4—C17—C18	122.6 (2)	C38—C43—H43	118.8
N4—C17—C16	121.0 (2)	N8—C44—C45	116.0 (3)
C18—C17—C16	116.2 (2)	N8—C44—H44A	108.3
C19—C18—C17	121.4 (2)	C45—C44—H44A	108.3
C19—C18—H18	119.3	N8—C44—H44B	108.3
C17—C18—H18	119.3	C45—C44—H44B	108.3
C18—C19—C14	122.5 (2)	H44A—C44—H44B	107.4
C18—C19—H19	118.8	C44—C45—C46	115.0 (3)
C14—C19—H19	118.8	C44—C45—H45A	108.5
N4—C20—C21	112.4 (2)	C46—C45—H45A	108.5
N4—C20—H20A	109.1	C44—C45—H45B	108.5
C21—C20—H20A	109.1	C46—C45—H45B	108.5
N4—C20—H20B	109.1	H45A—C45—H45B	107.5
C21—C20—H20B	109.1	C47—C46—C45	110.7 (3)
H20A—C20—H20B	107.9	C47—C46—H46A	109.5
C20—C21—C22	112.3 (3)	C45—C46—H46A	109.5
C20—C21—H21A	109.1	C47—C46—H46B	109.5
C22—C21—H21A	109.1	C45—C46—H46B	109.5
C20—C21—H21B	109.1	H46A—C46—H46B	108.1
C22—C21—H21B	109.1	C48—C47—C46	116.0 (3)
H21A—C21—H21B	107.9	C48—C47—H47A	108.3
C21—C22—C23	109.2 (2)	C46—C47—H47A	108.3
C21—C22—H22A	109.8	C48—C47—H47B	108.3
C23—C22—H22A	109.8	C46—C47—H47B	108.3
C21—C22—H22B	109.8	H47A—C47—H47B	107.4
C23—C22—H22B	109.8	N8—C48—C47	115.1 (3)
H22A—C22—H22B	108.3	N8—C48—H48A	108.5
C24—C23—C22	112.7 (3)	C47—C48—H48A	108.5
C24—C23—H23A	109.1	N8—C48—H48B	108.5
C22—C23—H23A	109.1	C47—C48—H48B	108.5
C24—C23—H23B	109.1	H48A—C48—H48B	107.5
			
C9—N1—N2—N3	−0.5 (3)	C17—N4—C24—C23	−160.3 (2)
C1—N1—N2—N3	−179.1 (2)	C20—N4—C24—C23	53.7 (3)
N1—N2—N3—C10	0.2 (3)	C22—C23—C24—N4	−54.0 (3)
C33—N5—N6—N7	0.6 (3)	C33—N5—C25—C26	127.7 (3)
C25—N5—N6—N7	178.6 (2)	N6—N5—C25—C26	−49.8 (3)
N5—N6—N7—C34	−0.3 (3)	C33—N5—C25—C30	−55.1 (4)
C9—N1—C1—C6	58.6 (4)	N6—N5—C25—C30	127.4 (3)
N2—N1—C1—C6	−123.2 (3)	C30—C25—C26—C27	−0.5 (4)
C9—N1—C1—C2	−122.4 (3)	N5—C25—C26—C27	176.7 (3)
N2—N1—C1—C2	55.8 (3)	C25—C26—C27—C28	0.2 (5)
C6—C1—C2—C3	−1.0 (4)	C26—C27—C28—C29	0.8 (5)
N1—C1—C2—C3	180.0 (3)	C26—C27—C28—C31	−177.1 (3)
C1—C2—C3—C4	−0.4 (5)	C27—C28—C29—C30	−1.7 (5)
C2—C3—C4—C5	1.1 (5)	C31—C28—C29—C30	176.3 (3)
C2—C3—C4—C7	179.9 (3)	C26—C25—C30—C29	−0.4 (4)
C3—C4—C5—C6	−0.4 (4)	N5—C25—C30—C29	−177.5 (3)
C7—C4—C5—C6	−179.2 (3)	C28—C29—C30—C25	1.5 (5)
C4—C5—C6—C1	−1.1 (4)	N6—N5—C33—C34	−0.6 (3)
C2—C1—C6—C5	1.8 (4)	C25—N5—C33—C34	−178.2 (2)
N1—C1—C6—C5	−179.2 (2)	N6—N5—C33—C32	176.9 (2)
N2—N1—C9—C10	0.6 (3)	C25—N5—C33—C32	−0.8 (4)
C1—N1—C9—C10	178.9 (2)	N6—N7—C34—C33	−0.1 (3)
N2—N1—C9—C8	−176.6 (2)	N6—N7—C34—C35	−178.2 (2)
C1—N1—C9—C8	1.8 (4)	N5—C33—C34—N7	0.4 (3)
N2—N3—C10—C9	0.2 (3)	C32—C33—C34—N7	−176.8 (3)
N2—N3—C10—C11	179.6 (2)	N5—C33—C34—C35	178.3 (3)
N1—C9—C10—N3	−0.4 (3)	C32—C33—C34—C35	1.1 (5)
C8—C9—C10—N3	176.4 (3)	N7—C34—C35—O2	177.9 (3)
N1—C9—C10—C11	−179.8 (2)	C33—C34—C35—O2	0.2 (4)
C8—C9—C10—C11	−3.0 (5)	N7—C34—C35—C36	0.0 (4)
N3—C10—C11—O1	−178.9 (2)	C33—C34—C35—C36	−177.6 (3)
C9—C10—C11—O1	0.4 (4)	O2—C35—C36—C37	−9.2 (5)
N3—C10—C11—C12	2.5 (4)	C34—C35—C36—C37	168.5 (3)
C9—C10—C11—C12	−178.2 (2)	C35—C36—C37—C38	−177.2 (3)
O1—C11—C12—C13	6.3 (4)	C36—C37—C38—C43	177.0 (3)
C10—C11—C12—C13	−175.1 (2)	C36—C37—C38—C39	−3.3 (5)
C11—C12—C13—C14	−178.5 (2)	C43—C38—C39—C40	−0.4 (5)
C12—C13—C14—C19	178.4 (2)	C37—C38—C39—C40	179.8 (3)
C12—C13—C14—C15	0.0 (4)	C38—C39—C40—C41	0.8 (6)
C19—C14—C15—C16	−0.9 (4)	C48—N8—C41—C42	8.7 (4)
C13—C14—C15—C16	177.7 (2)	C44—N8—C41—C42	155.8 (3)
C14—C15—C16—C17	0.6 (4)	C48—N8—C41—C40	−174.0 (3)
C24—N4—C17—C18	2.9 (4)	C44—N8—C41—C40	−27.0 (4)
C20—N4—C17—C18	146.9 (2)	C39—C40—C41—C42	−0.2 (5)
C24—N4—C17—C16	178.4 (2)	C39—C40—C41—N8	−177.6 (3)
C20—N4—C17—C16	−37.7 (3)	N8—C41—C42—C43	176.6 (3)
C15—C16—C17—N4	−175.2 (2)	C40—C41—C42—C43	−0.8 (5)
C15—C16—C17—C18	0.5 (4)	C41—C42—C43—C38	1.3 (5)
N4—C17—C18—C19	174.4 (2)	C39—C38—C43—C42	−0.7 (4)
C16—C17—C18—C19	−1.2 (3)	C37—C38—C43—C42	179.1 (3)
C17—C18—C19—C14	1.0 (4)	C41—N8—C44—C45	168.4 (3)
C15—C14—C19—C18	0.1 (4)	C48—N8—C44—C45	−43.2 (4)
C13—C14—C19—C18	−178.5 (2)	N8—C44—C45—C46	46.0 (5)
C17—N4—C20—C21	159.5 (3)	C44—C45—C46—C47	−45.7 (5)
C24—N4—C20—C21	−54.4 (3)	C45—C46—C47—C48	45.5 (5)
N4—C20—C21—C22	54.4 (4)	C41—N8—C48—C47	−169.6 (3)
C20—C21—C22—C23	−52.9 (4)	C44—N8—C48—C47	42.2 (5)
C21—C22—C23—C24	52.9 (4)	C46—C47—C48—N8	−44.9 (5)

### Hydrogen-bond geometry (Å, º)

Cg1 is the centroid of the C14–C19 benzene

**Table:**

*D*—H···*A*	*D*—H	H···*A*	*D*···*A*	*D*—H···*A*
C6—H6···O1^i^	0.93	2.51	3.415 (3)	165
C3—H3···*Cg*1^ii^	0.93	2.73	3.469 (3)	137

Symmetry codes: (i) −*x*+1, −*y*+1, −*z*+1; (ii) *x*+1, *y*, *z*.
